# Development and characterisation of highly antibiotic resistant *Bartonella bacilliformis* mutants

**DOI:** 10.1038/srep33584

**Published:** 2016-09-26

**Authors:** Cláudia Gomes, Sandra Martínez-Puchol, Lidia Ruiz-Roldán, Maria J. Pons, Juana del Valle Mendoza, Joaquim Ruiz

**Affiliations:** 1 ISGlobal, Barcelona Ctr. Int. Health Res. (CRESIB), Hospital Clínic - Universitat de Barcelona, Barcelona, Spain; 2 School of Medicine, Research Center and Innovation of the Health Sciences Faculty, Universidad Peruana de Ciencias Aplicadas (UPC), Lima, Peru; 3 Instituto de Investigación Nutricional, Lima, Peru

## Abstract

The objective was to develop and characterise *in vitro Bartonella bacilliformis* antibiotic resistant mutants. Three *B. bacilliformis* strains were plated 35 or 40 times with azithromycin, chloramphenicol, ciprofloxacin or rifampicin discs. Resistance-stability was assessed performing 5 serial passages without antibiotic pressure. MICs were determined with/without Phe-Arg-β-Napthylamide and artesunate. Target alterations were screened in the *23S rRNA*, *rplD*, *rplV*, *gyrA*, *gyrB*, *parC, parE* and *rpoB* genes. Chloramphenicol and ciprofloxacin resistance were the most difficult and easiest (>37.3 and 10.6 passages) to be selected, respectively. All mutants but one selected with chloramphenicol achieved high resistance levels. All rifampicin, one azithromycin and one ciprofloxacin mutants did not totally revert when cultured without antibiotic pressure. Azithromycin resistance was related to L4 substitutions Gln-66 → Lys or Gly-70 → Arg; L4 deletion Δ_62–65_ (Lys-Met-Tyr-Lys) or L22 insertion 83::Val-Ser-Glu-Ala-His-Val-Gly-Lys-Ser; in two chloramphenicol-resistant mutants the *23S rRNA* mutation G2372A was detected. GyrA Ala-91 → Val and Asp-95 → Gly and GyrB Glu474 → Lys were detected in ciprofloxacin-resistant mutants. RpoB substitutions Gln-527 → Arg, His-540 → Tyr and Ser-545 → Phe plus Ser-588 → Tyr were detected in rifampicin-resistant mutants. In 5 mutants the effect of efflux pumps on resistance was observed. Antibiotic resistance was mainly related to target mutations and overexpression of efflux pumps, which might underlie microbiological failures during treatments.

*Bartonella bacilliformis* is the causative agent of Carrion’s disease, a biphasic endemic illness of the Andean valleys. In the acute stage (the so-called Oroya fever) severe haemolytic anaemia is present, resulting in 40–85% of deaths in untreated people and decreases to around 10% if correctly treated^1^,^2^,^3^. In this stage the presence of concomitant infections such as bloodstream *Salmonella* infections, among others^1^,^4^,^5^,^6^, are frequent due to the temporal immunosuppression induced by *B. bacilliformis*^7^.

Peruvian wart is the chronic non life-threatening phase, characterised by cutaneous proliferative vascular lesions, occurring some weeks or months after the acute infection^3^.

Additionally, the number of asymptomatic carriers is uncertain, although some studies have shown that 45% of the inhabitants of endemic areas present evidence of previous contact with the pathogen^8^.

The usual treatments are chloramphenicol (CHL) or ciprofloxacin (CIP), alone or combined with cephalosporins or aminoglycosides in for the acute stage. Rifampicin (RIF) or azithromycin (AZM) are used in the chronic stage^6^,^9^. Despite the reported ~10% of deaths among patients receiving adequate treatment^3^, it is widely considered that this microorganism has good clinical response to the above mentioned treatments.

The use of antibacterial agents in the treatment of Carrion’s disease has been profuse in recent decades^10^. Moreover, in asymptomatic carriers the microorganisms are also under antibiotic pressure related to the treatment of other infections. However, descriptions of antibiotic-resistant *B. bacilliformis* clinical isolates are scarce^11^,^12^. To date, only constitutive nalidixic acid resistance and related diminished fluoroquinolone susceptibility have been reported in association with the presence of an Ala as WT amino acids at positions 91 and 85 of GyrA and ParC, respectively^13^,^14^. These characteristics are extended to other members of the *Bartonella* genus^13^. Additionally, relatively high Minimal Inhibitory Concentration (MIC) levels of clindamycin and colistin^12^, have been observed as well as sporadic isolates presenting resistance to CHL or CIP and a trend towards diminished susceptibility to aminoglycosides^11^,^16^. Moreover, *in vitro* resistance to different antimicrobial agents, including coumermycin, CIP, RIF and erythromycin, has been described^17^,^18^,^19^. However, these studies were developed using either the KC583 or KC584 strain alone, being limited to the analysis of point mutations, and to date, no study has determined the role of efflux pump overexpression or the stability of the antibiotic resistance selected.

Analysis of *in vitro* obtained mutants may provide information in order to better understand antibiotic-resistance acquisition and evolution. The aim of this study was to develop and characterise a series of *in vitro B. bacilliformis* antibiotic resistant mutants and determine the presence of target mutations, the role of efflux pumps as well as the stability of selected resistance.

## Results

### Development of antibiotic-resistant mutants

**Figure 1:**
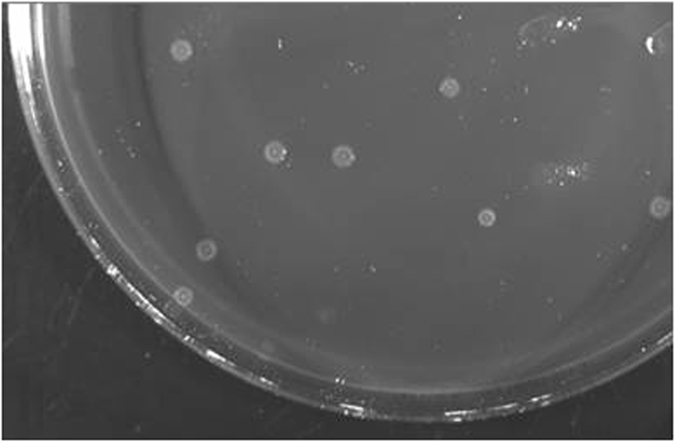
Colony morphology. The photograph shows *B. bacilliformis* presenting T1 colony morphology^20^. The colony is characterised by a small, translucent round morphology, with a regular edge and a small halo. The colonies present a “bubble” in the center of the colony. The morphology was unstable and disappeared after reculture.

The time required for bacterial lyophilised reactivation varied from 5 weeks (strains 57.19 and 57.20) to 9 weeks (strain 57.18). Interestingly, strain 57.18 showed an initially different morphology, coinciding with the previously described T1 morphology^20^, although this reverted in the next passage (Fig. 1).

The development of the antibiotic-resistant mutants required approximately 18 months, therefore, 4 antibiotic-resistant mutants were obtained from each parental strain, one for each antibiotic included in the study.

**Figure 2:**
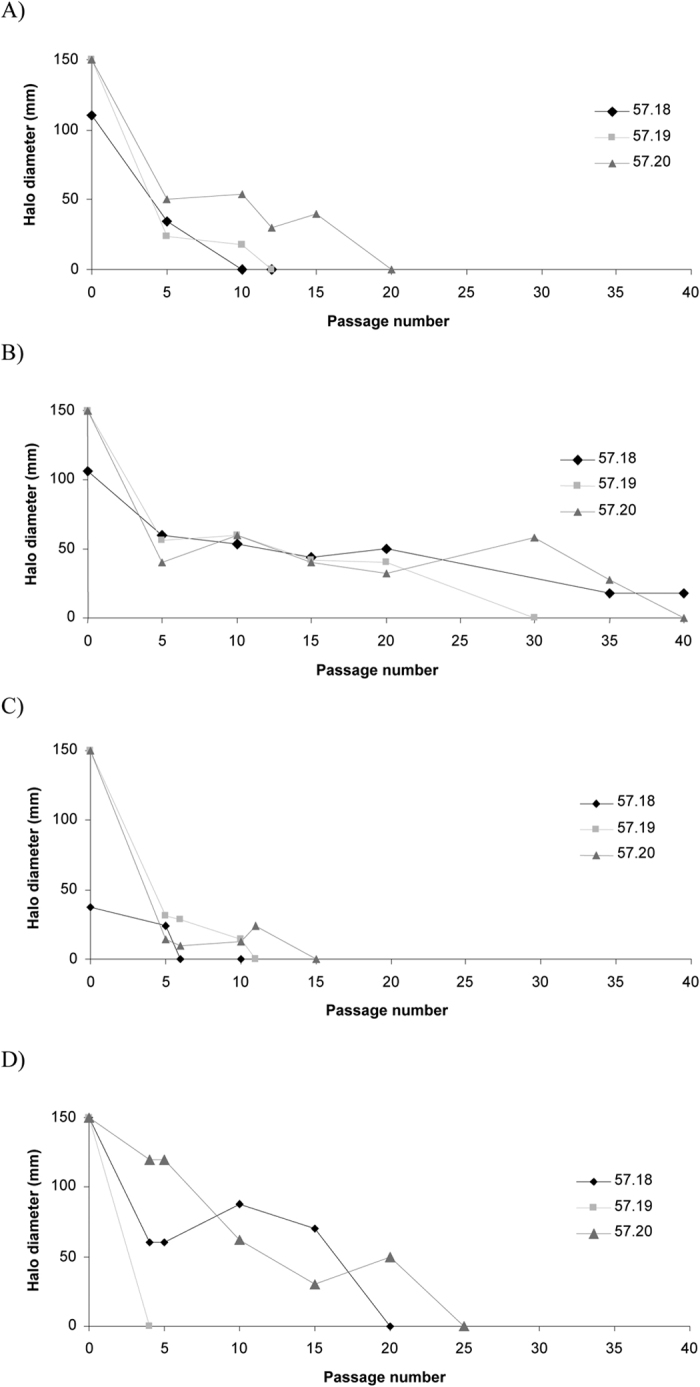
Evolution of disc diameter halo during serial passages. (**A**) Azithromycin, (**B**) Chloramphenicol, (**C**) Ciprofloxacin, (**D**) Rifampicin. This figure demonstrates the ease with each mutant are selected for each antibiotic. The halo diameters (measured in mm) are reported every 5 passages or at the passage in which halo zero was obtained. In (**B**) is clearly visualised the difficulty with which resistance to chloramphenicol (CHL) is developed.

The first antibiotic to generate inhibitory halo 0 mm was RIF, with only 4 passages to obtain confluent growth (strain 57.19). However, overall, the antibiotic requiring the least number of passages to generate confluent growth was CIP with a mean of 10.6 passages. On the other hand, CHL required >37.3 passages (Table 1). Thin growth was observed inside the halo recorded during the process of mutant selection of 57.20_Azm_. Thus, after the initial 35 passages, most antibiotic-resistant mutants showed confluent growth in the presence of the antibiotic disc (inhibitory halo 0 mm) except for two out of three mutants selected with CHL, which presented inhibitory halos of 18 mm (57.18_Chl-35_) and 32 mm (57.20_Chl-35_). After 5 additional serial passages (total: 40 passages), 57.20_Chl-40_ achieved a halo of 0 mm, while 57.18_Chl-40_ remained with an inhibitory halo of 18 mm (Fig. 2).

### Analysis of MIC levels and stability of resistance

A maximum of 1 dilution difference between the MICs at 7 and 14 days was observed. Thus, those obtained at 7 days were used throughout the manuscript.

Analysis of the MIC levels showed that all but 1 mutant achieved MICs up to the E-test detection limit >256 mg/L (AZM, RIF and CHL) and >32 mg/L (CIP). The exception was the above mentioned 57.18_Chl-40_ mutant that reached a CHL MIC level of 4 mg/L (Table 2).

Interestingly, analysis of the stability of the resistance obtained showed that all RIF selected mutants were stable, as were 57.18_Azm-5St_ and 57.18_Cip-5St_. Two strains (57.18_Chl-5St_ and 57.19_Chl-5St_) returned to the parental MIC levels, while the remaining isolates showed intermediate MICs between parental and final mutant values. In the case of mutants 57.19_Azm-5St_ and 57.20_Cip-5St_ at 14 days the presence of colonies within the inhibitory halo were observed. Thus, 57.19_Azm-5St-WH_ showed a MIC of AZM of 32 mg/L while 57.20_Cip-5St-WH_ showed a MIC > 32 mg/L for CIP (Table 2).

### Antibiotic cross resistance

When the mutants were selected with AZM the MICs to other antibiotics generally increased, while the selection with CHL usually resulted in decreases in other MICs, and in those selected with CIP and RIF the results were more variable (Tables 2 and 3).

### Target mutations

None of the genes of the parental strains analysed presented differences with respect those of the KC583 strain except the amino acid codon change Thr-13 → Ala at L4 protein and Arg-9 → Cys at L22 protein.

AZM resistance was related to the presence of alterations in the *rplD* and *rplV* genes, encoding the L4 and L22 proteins, respectively. No mutation was observed in the *23S rRNA* gene. Thus 57.18_Azm_ and 57.18_Azm-5st_ showed a predicted 4 amino acid deletion (Lys-Met-Tyr-Lys) at L4 protein from codons 62 to 65 (Δ_62–65_). Nonetheless, close analysis of the spherogram showed the presence of a double sequence from the deletion onwards under the majority peaks with in the reading-frame together with a non-deleting *rplD* (Supplementary Figure 1). Meanwhile, in mutants 57.19_Azm-35_ and 57.19_Azm-5st_ we observed the presence of the L4 amino acid codon change Gly-70 → Arg, with a change His-74 → Tyr also being observed in 57.19_Azm-5st-WH_. The mutant 57.20_Azm_ showed the presence of alterations at both the *rplD* and *rplV* genes. Thus, the presence of an amino acid codon change Gln-66 → Lys was observed in the *rplD* gene, while a 27 base-pair insertion (leading to the 9 amino acid Val-Ser-Glu-Ala-His-Val-Gly-Lys-Ser) was observed at one of the two *rplD B. bacilliformis* genes after position 83 (Supplementary Figure 1). Interestingly the 57.20_Azm-5St_ *rplD* gene showed a double peak which presented a mixed population with a partial reversion of the change Gln-66 → Lys.

**Figure 3:**
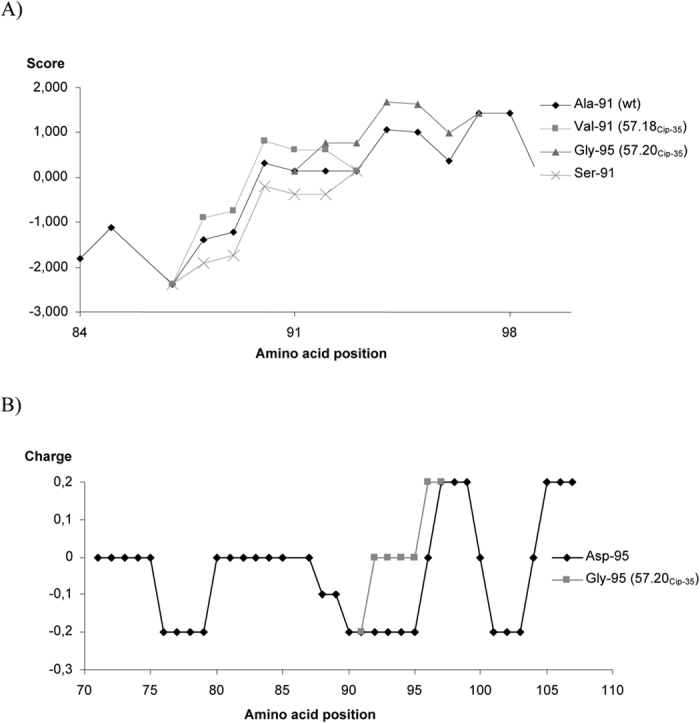
Hydrophobic and charge patterns associated with GyrA amino acid substitutions. (**A**) Alterations in the hydrophobic pattern. Additionally to the amino acid substitutions detected (Ala-91 → Val and Asp-95 → Gly) the theoretical effect of the presence of Ser-91 also shows the gradual effect on the hydrophobic pattern related to the presence of Ser, Ala or Val at position 91. (**B**) Effect on the charge pattern. This graph only shows the effect of Asp-95 and Gly-95 since the presence of Val-91 does not result in charge pattern alterations. The (**A**) comprises the amino acid sequence from amino acids 84 to 99, while in (**B**) the amino acid sequences analysed are from amino acids 70 to 107.

Regarding quinolone resistance, no mutation was observed in the Topoisomerase IV encoding genes (*parC* and *parE*). A GyrA amino acid substitution was observed in 2 out of 3 selected mutants, 57.18_Cip-35_ and 57.20_Cip-35_ (Ala-91 → Val and Asp-95 → Gly, respectively), and a GyrB amino acid substitution (Glu-475 → Lys) was observed in 57.19_Cip-35_. The mutations present in 57.18_Cip-35_ and 57.19_Cip-35_ were stable and were also observed in the mutants 57.18_Cip-5St_, and 57.19_Cip-5St_. In the case of 57.20_Cip-35_ the analysis of the sequence spherogram showed the presence of a double peak with two bacterial populations, one with the WT *gyrA* gene sequence and other possessing the aforementioned amino acid codon substitution. This was confirmed by analysing 57.20_Cip-5St_ (possessing a WT *gyrA* gene sequence) and 57.20_Cip-5St-WH_ (which maintained the amino acid change Asp-95 → Gly). The *in silico* determination of the hydrophobicity pattern showed an alteration in the presence of GyrA Val-91, while the presence of GyrA Asn-95 altered both the charge and slightly affected the hydrophobicity pattern (Fig. 3).

In the 57.19_Chl-35_ mutant an alteration in the *23S rRNA* gene (G2372A) was detected, which reverted after 5 passages without antibiotic pressure. The same mutation was also observed in the 57.20_Chl-40_ mutant, but a double peak was observed showing the presence of a double bacterial population, or, more probably, that the mutation was only present in one of the two *23S rRNA* genes of the *B. bacilliformis* genome (Supplementary Figure 1).

Finally, the presence of mutations in the *rpoB* gene was observed in all RIF-selected mutants which were also present after the 5 passages without antibiotic pressure, leading to the amino acid changes Gln-527 → Arg (57.18_Rif-35_, 57.18_Rif-5St_), His-540 → Tyr (57.19_Rif-35_, 57.19_Rif-5St_) and Ser-545 → Phe plus Ser-588  → Tyr (57.20_Rif-35_ and 57.20_Rif-5St_). Interestingly, when the *rpoB* gene in the 57.20 RIF-derived mutant at passage 13 (57.20_Rif-13_), was sequenced, only the presence of the amino acid codon change Ser-588  → Tyr was observed.

### Effect of Efflux Pumps Inhibitors (EPIs)

The study of the effect of Phe-Arg-β-Napthylamide (PAβN) and artesunate (ART) on normal bacterial growth showed that high PAβN concentrations (7.5, 10, 20 mg/L) allowed bacterial, albeit non normal, growth. Thus, a concentration of 5 mg/L was used in the assays. No effect of ART was observed on bacterial growth with 20 mg/L. Additionally, we confirmed the lack of effect on bacterial growth with ethanol at the concentration and required volume used.

Both PAβN and ART had a visible effect on the CHL MIC of 57.18_Chl-40_, and this effect was also confirmed by the increased disc inhibitory halo in the presence of both EPIs. Both EPIs also affected the AZM susceptibility levels of the 57.18_Azm-35_ mutant. Additionally, ART was found to affect the RIF MIC of 57.19_Rif-35_, and also enhanced the activity of RIF and CIP in 57.20_Cip-35_ and 57.20_Rif-35_ mutants respectively (Tables 2 and 4).

## Discussion

Currently antimicrobial resistance levels have only been established in a few *B. bacilliformis* clinical or collection isolates^11^,^12^,^14^,^15^,^16^. Information about the mechanisms of antibiotic resistance exhibited by these isolates is very scarce and strictly focused on constitutive quinolone resistance^13^,^14^,^15^. This lack of data, together with the high percentage of clinical cure without microbiological elimination described in more than 22% of CIP and up to 50% of CHL treatments as well as the high-lethality related to the acute illness phase in the absence or delay of treatment^2^,^3^,^21^, highlight the need for *in vitro* information about the ease with which this microorganism develops resistance and which antibiotic resistance mechanisms are selected. To the best of our knowledge only three *in vitro* studies has been developed to date^17^,^18^,^19^, and none has determined the stability of the antibiotic resistance selected or the role of efflux pump overexpression.

Although the 57.19 strain only needed 4 consecutive passages to develop full resistance to RIF, overall, the antibiotic which most easily selected resistant mutants was CIP. This is in accordance with the possession of Ala-91 and Ala-85 of GyrA and ParC respectively which affect the hydrophobicity pattern of these proteins, impairing the interaction of quinolones and acting as a factor favouring the selection of resistance to fluoroquinolones^14^,^22^. In fact, it has been shown that the frequency of mutation of *B. bacilliformis* KC583 was 10-fold higher than that of *Escherichia coli*, while only 5 passages were needed to select high CIP-resistant mutants from strain KC584^18^,^19^. Regarding RIF and AZM, full resistance was obtained after 3 and 4 passages respectively^18^. There are no previous reports on the selection of *Bartonella* spp. CHL-resistant mutants. In our study, resistance to CHL was consistently the most difficult to obtain. Although this finding is in apparent disagreement with the observed 50% of persistent bacteraemia after CHL treatments^21^, it might be related to changes at the fitness level as has been observed in the presence of specific *23S rRNA* gene point mutations in other microorganisms^23^,^24^,^25^.

The MIC of the mutants selected was higher than that of the parental isolates, achieving high MICs levels with all the antibacterial agents tested. This finding shows a worrisome scenario: the feasibility of selecting high antibiotic resistant mutants with the 4 main antibiotic families used in the treatment of Carrion’s disease. However, only those mutants selected with RIF showed consistent resistance stability, while only 1 out of 3 of CIP or AZM-resistant mutants did not revert either totally or partially. Regarding CHL, all the resistant mutants selected reverted, supporting the idea of a high biological cost of the development of CHL resistance. In the same line, the presence of double sequences when analysing some of antibiotic target gene sequences may be interpreted in three different ways: The survival of original susceptible parental strains, the partial reversion of the mutants obtained when antibiotic pressure disappears or the presence of mutations in only one of the gene copies when there is more than one. The first possibility is unlikely because of the high susceptibility levels of the parental strains to all the antibacterial agents tested (halo diameter >100 mm) which prevent the survival of the microorganisms when the disc was placed on the center of the plate (the maximum possible halo diameter is 50 mm). Meanwhile the other two possibilities may be considered, and, moreover, are not mutually exclusive. They might underlie the high number of microbiological failures, isolation of viable microorganisms from blood, after successful clinical treatments^21^. Moreover, the reversion observed in the 57.20_Azm-35_ mutant may be related to the slower growth described in association with L4 and L22 alterations^26^. Along this line, the reversion of resistance, the presence of double bacteria populations in at least two mutants (one selected with CIP and other with AZM) as well as the additional time needed by some of the resistant strains (57.19_Azm-5St-WH_, 57.20_Cip-5st-WH_) to grow, support the idea that the development of antibiotic resistance has a high biological cost in *B. bacilliformis*, resulting in the dilution of strains carrying specific antibiotic-related mutations within a general antibiotic susceptible bacterial population. Similarly, both the loss of biological efficiency and the decrement of virulence after the development of antimicrobial resistance have been observed in other microorganisms^27^,^28^. However, compensatory mutations might result in the development and spread of resistant isolates^27^,^29^.

Although non-uniform, cross effects, either increases or decreases, in the MIC levels were observed. These alterations might be explained by collateral antibiotic resistance effects that could interfere with normal bacteria biochemical processes. Three of antibacterial agents tested act at a ribosome level, while the remaining agent acts at the proteins involved in DNA decatenation and duplication. Ribosomal level alterations may differentially affect the expression of genes or may have a direct effect on the activity of other agents. In other microorganisms, specific points placed in *23S rRNA* and in the ribosomal proteins may result in concomitant resistance between CHL and macrolides^30^,^31^. Among these, it has been suggested that L4 deletions Δ_65–66_ (Trp-Arg) and Δ_68–69_ (Lys-Gly) leading to macrolide-resistance in *Streptococcus pneumoniae* also confres low levels of CHL resistance. Thus, *S. pneumoniae* L4 Δ_65–66_ has been associated with an increase of the CHL MIC of up to 4-fold when cloned in a pan-susceptible *S. pneumoniae* strain^31^. Amino acid codons 65 and 66 of *S. pneumoniae* are equivalent to positions 64 and 65 of *B. bacilliformis*, both included within the Δ_62–65_ of 57.18_Azm-35_ mutant. Thus, the role of L4 deletions in the acquisition of the low levels of CHL resistance observed in this mutant (MIC 2 mg/L, 5.26-fold increases) is plausible, either directly due to alterations in the ribosome conformation affecting CHL binding or indirectly due to the possible induction of efflux pump overexpression as discussed below. This fact highlights the risk of co-selection of antibiotic resistance during *B. bacilliformis* treatment.

On the other hand, selection of CHL seems to negatively affect the ability of bacteria to survive in the presence of other antibiotic agents. This fact may also be related to the above mentioned possible effect on fitness, being probably underlain by alterations in the expression or regulation of different bacterial genetic factors. Additionally, selection of hypersusceptibility to unrelated antibiotics, such as macrolides, has also been observed in relation to specific *23S rRNA* mutations^32^.

Two differences leading to the substitutions Thr-13 → Ala at L4 and Arg-9 → Cys at L22, were observed between the parental and KC583 strains. Nonetheless, both substitutions were present in other *B. bacilliformis* genomes. This finding, together with the high antibiotic susceptibility of the parental isolates, suggests the presence of DNA polymorphisms.

Regarding AZM resistance, in all the cases the presence of alterations at L4 and L22 was found, while no mutation at *23S rRNA* was observed. This latter finding differs from that described in microorganisms with a low number of *23S rRNA* gene copies^33^ and observed in previous studies developed in *B. bacilliformis* and *Bartonella henselae*^18^,^34^,^35^.

Amino acid changes at L4 and L22, including insertions and deletions, have been described in a series of unrelated microorganisms, such as *E. coli*^26^,^36^,^37^. Regarding *Bartonellaceae*, the mutations at L4 (Gly-70 → Arg and Gly-70 → Arg + His-74 → Tyr) have been previously described in *in vitro* obtained macrolide-resistant mutants of *B. henselae*^35^. In *E. coli*, a series of amino acid changes involving the L4 residues Gln-62 and Gly-66 equivalent to *B. bacilliformis* Gln-66 and Gly-70 have been reported as being involved in the development of AZM resistance^26^,^36^. Interestingly, in *E. coli* substitutions at position Ser-70 (equivalent to *B. bacilliformis* His-74) have been described^36^, but never alone, as reported by Biswas *et al*.^34^, suggesting a minor role of this substitution in the development of macrolide resistance.

This is the first report describing insertions and deletions in the L4 and L22 proteins of *B. bacilliformis*. The predicted L4 deletion is located close to the L4 “hot spot” region involved in macrolide resistance in which a deletion of the 2 equivalent later amino acids in macrolide-resistant *S. pneumoniae* has been reported^31^, while to the best of our knowledge no insertion has been described in equivalent positions in other microorganisms.

Two out of three CHL-resistant mutants (57.19_Chl-35_ and 57.20_Chl-40_) presented a G2372A mutation in the *23S rRNA* gene. The equivalent mutation has previously been involved in CHL resistance in yeasts and *E. coli*^25^,^38^. Although in the case of the 57.20_Chl-40_ mutant double peaks were obtained suggesting either the presence of a double bacterial population, or, more probably, the presence of a mutation affecting only one of the two *23S rRNA* genes. This fact, together with the rapid reversion of this mutation and the slow bacterial growth, which might be longer than 2 months in clinical samples cultures^10^,^39^, might explain the presence of microbiological failures after CHL treatments, contributing to the lack of reports of CHL resistance in clinical isolates. Supporting the biological cost of the development of CHL resistance, the equivalent G2447A mutation in *E. coli* results in both resistance to CHL and in retarded growth rates^25^.

The amino acid alterations observed in two out of three GyrA were located in the classical quinolone-DNA Gyrase interaction points, while resistance in the third isolate was related to the presence of mutations in the *gyrB* gene. Up to now, no amino acid change at GyrA position 91 has been described in *B. bacilliformis*. However, alterations at equivalent GyrA position have been extensively described in other quinolone-resistant microorganisms^22^, including the presence of Val-91, detected both in *B. henselae* and unrelated microorganisms^22^,^34^,^40^. The presence of an Ala in position 83 of the GyrA of *E. coli* (equivalent to 91 of *B. bacilliformis)* results in a decreased CIP MIC, and intermediate or low-level of nalidixic resistance^40^,^41^,^42^, while the presence of Val results in full fluoroquinolone-resistance as well as in high levels of resistance of nalidixic acid^40^. These findings may be directly related to the different effect on the GyrA hydrophobicity pattern and the subsequent involvement of the ability of quinolones to bind with their targets in relation to the presence of Ser (WT amino acid in *E. coli* and most other microorganisms) Ala or Val residues, as shown in Fig. 3.

Changes at position Asp-95, including Asp-95 → Gly, have been previously described in studies analysing quinolone-resistant mutants of *B. bacilliformis*, *B. henselae* and *Bartonella quintana*^18^,^19^,^43^. Changes at equivalent position have frequently been reported in other microorganisms^22^,^40^. The effect of different mutations is additive^22^, thus, in the absence of the effect on bacterial fitness, it would be more successful to add a change in a new position than to vary the amino acid at position 91. The presence of Gly at position 95 slightly affects the hydrophobicity pattern, but mainly affects the pattern of charges at this area, avoiding the interaction with radical 7 of the quinolones^44^.

This is the first description of a GyrB amino acid substitution Glu-475 → Lys in *B. bacilliformis*. The same amino acid change at the equivalent position of *S. pneumoniae* (Glu-474) has been associated with quinolone resistance^45^. Moreover, in *S. pneumoniae* a series of amino acid changes including Glu-476 → Ala and Arg-477 → His has also been described in the same region, showing the relevance of the GyrB region in the development of quinolone-resistance^46^,^47^. The 57.19_Cip-5St_ mutant showed a MIC of 1.5 mg/L (3.95-fold higher than its parental isolate). Thus, by itself the Glu-475 → Lys alteration results in a slight increase in the CIP MIC. Moreover, this finding, together with the lack of PAβN and ART effect on the CIP MIC, highlight the presence of unstable mechanism/s of resistance in the 57.19_Cip-35_ mutant.

Rifamycin-resistant *rpoB* gene mutations are usually located within 3 highly conserved regions in the mid portion of the gene. In *E. coli* these regions include codons 505–537 (cluster I), 563–575 (cluster II) and 684–690 (cluster III)^48^,^49^. In the present study, the amino acid codon changes Gln-527 → Arg; His-540 → Tyr; Ser-545 → Phe are located within the *B. bacilliformis* equivalent cluster I, while Ser-588 → Tyr is located within the equivalent cluster II. Of these, the amino acid change Ser-545-Phe has been previously observed in *B. bacilliformis* and *B. quintana in vitro* RIF-resistant mutants^18^. However, substitutions at equivalent amino acid positions have been observed as being involved in the development of rifamycin resistance in other microorganisms including *Mycobacterium tuberculosis*^50^. Thus, in *E. coli* RpoB substitutions of Gln-513, His-526, Ser-531 or Ser-574 (equivalents to Gln-527, His-540, Ser-545 and Ser-588 of *B. bacilliformis* respectively) have been described; moreover, in some cases the same amino acid change (e.g. Ser-531 → Phe or His-526 → Tyr) has also been reported^43^,^51^,^52^. In one case (57.20_Rif-35_) a double mutation (Ser-545 → Phe plus Ser-588 → Tyr) was observed. However, when the presence of an RpoB alteration was sought in 57.20_Rif_ at passage 13, only the presence of the amino acid change Ser-588 → Tyr was observed. This fact shows the sequential selection of RIF resistance related to continuous antibiotic pressure. In *E. coli* the substitution at Ser-574 (equivalent to Ser-588) results in low-levels of RIF resistance, being its effect strongly diminished in the presence of PAβN^51^. Thus, the selection of a mutation conferring low RIF resistance levels is followed by that of another additive mechanism of resistance, leading to an increase in the RIF resistance levels. Similarly, although substitutions at the equivalent Ser-545 position seem to result in high RIF MIC levels in *E. coli*^49^, in other microorganisms such as *Rhodococcus equi* only low levels of RIF resistance are conferred^53^.

This is the first determination of the role of efflux pumps in the development of antibiotic resistance in *B. bacilliformis*. The two EPI tested act differently, while PAβN competes with other efflux pump substrates to be extruded from bacteria, ART diminishes the expression levels of some efflux pump encoding genes^54^,^55^. These differences may underlie the disparity of results obtained in the present study with these inhibitors.

Both PAβN and ART affected the susceptibility levels of 57.18_Azm-35_ and 57.18_Chl-40_ mutants. In the first case the mutant presented L4 Δ_62–65_. Although related to deletions in L22, a similar scenario has been observed in *E. coli* strains carrying 3 amino acid deletions in L22 in which the inactivation of different efflux pump components results in a strong decrease in the erythromycin MIC levels^56^. Thus, it has been proposed that deletions at L22 alter the translation of specific proteins, possibly via changes in programmed stalling, modifying the cell envelope and resulting in efflux pump overexpression and lowering steady-state macrolide levels^56^. It is of interest to note that in 57.18_Azm-35_, the use of PAβN was only visible in the disc diffusion analysis. The most probable scenario for this is that the MIC of this strain is much higher than 256 mg/L and thus, the effect on the MIC levels, albeit present, was not observed. Regarding the 57.18_Chl-40_ mutant both the effect of PAβN and ART decreased the CHL MIC levels to only slightly higher than the parental isolate, showing that efflux pumps may play a role in the development of low levels of CHL resistance in *B. bacilliformis*. No effect was observed with any inhibitor tested in either 57.19_Chl-35_ or 57.20_Chl-40_. This finding, together with the effect of ART on the antimicrobial susceptibility levels of all remaining 57.20 derivative mutants, highlight two facts: (a) the high levels of resistance to CHL derived from the presence of *23S rRNA* mutations which when present mask the effect of efflux pumps, and (b) the presence of efflux pumps able to extrude the 4 antimicrobial families tested in *B. bacilliformis*. Thus, the different level of antimicrobial resistance of each selected mechanism may allow or not visualization of the overexpression of efflux pumps and might underlie the differences in the effect of ART on the RIF-selected mutants. ART also affects the susceptibility to RIF in 57.19_Rif-35_ and 57.20_Rif-35_ mutants, showing that efflux pumps may also affect this antibiotic in *B. bacilliformis* and reinforcing the possibility that the selection of a double RpoB substitution in the 57.20_Rif-35_ mutant was due to the low level of RIF resistance conferred. In the case of the 57.19_Rif-35_ mutant the effect was only observed on the MIC levels, while in the case of 57.20_Rif-35_ the effect was observed in the disc diameter halo. Further studies are needed to determine the mechanisms of efflux pump overexpression (alterations in signal patterns, punctual mutations in promoter regions, among others).

In summary, the present data highlight the ability of *B. bacilliformis* to become antibiotic resistant both by the development of antibiotic-target alterations, but also mediated by efflux pump overexpression. However, regarding AZM, CHL and CIP, the instability of resistance detected in different mutants, either related to reversions and/or to dilution within a higher fitness susceptible population, suggests a high biological cost, which may underlie the rarity of antibiotic-resistant *B. bacilliformis* clinical isolates. Further studies analysing antibiotic target sequences from a relevant number of *B. bacilliformis* clinical isolates prior, during and after antibiotic treatment are necessary to better understand the natural history of antibiotic resistance in this microorganism.

## Methods

### Microorganisms

Three *B. bacilliformis* strains from the Institute Pasteur (Paris, France) collection were used (Table 5). The strains were reactivated following the instructions of the Institute Pasteur. Briefly, after opening, 0.2 ml of LB was added to the vial to resuspend the bacteria. Thereafter, the suspension was spread onto Columbia blood agar (Ref: 254005, BD, Heidelberg, Germany) and incubated at 28 °C. The plates were inspected weekly until bacterial growth was observed. Prior to the development of the antibiotic resistant mutants, the bacterial identity of all three isolates was confirmed by amplification and sequencing of the *16S rRNA* gene (Table 6)^59^.

### In vitro development of resistant mutants

The antibiotic resistant mutants were selected by plating the *B. bacilliformis* on blood agar with a disc of AZM (15 μg), CIP (5 μg), CHL (30 μg) or RIF (30 μg) (BD, Franklin Lakes, USA) which was initially put in the corner of each plate, and subsequently in the center of the plate according to halo diameter^18^. The confluent growth outside the zone of inhibition was recovered with a pre-sterilized plastic inoculation loop and subcultured 35 consecutive times. In those cases in which no halo zero was obtained, the isolates were grown in the presence of antibiotic for 5 additional passages.

At the beginning of the study, the passages with the selected antibiotic discs were performed every other week because of the difficulty to visualise the bacterial growth in the first weeks due the large halo sizes. Then, we changed to a weekly basis, according to the evolution of each strain.

Throughout the text, the mutants obtained are referred to by indicating the name of the parental isolate, the antibiotic used in its selection and the passage number (e.g.: 57.18_Azm-35_ is the AZM resistant mutant derived from strain 57.18 at passage 35).

### Stability of resistance

Resistance stability was assessed by doing 5 final additional passages on Columbia blood agar, on a weekly basis, in the absence of antibiotic pressure. These are referred to in the text following the above mentioned nomenclature but including “5st” (5 serial passages to determine stability instead of passage number). When a mixed bacterial population was obtained (e.g.: growth of isolated colonies inside the inhibitory halo), isolates from inside of halo were additionally marked as “WH” (within halo).

### Antibiotic susceptibility levels

The MICs of AZM, CIP, CHL and RIF of the parental isolates, the mutants at the final passage and after the 5 additional passages without antibiotic were determined by E-test (Liofilchem, IZASA, Barcelona, Spain). The MICs were read at 7 and 14 days. To determine the effect on the MIC levels the quotient MIC_F_ (final MIC)/MIC_I_ (initial MIC) was used. In all cases MIC differences >2-fold were considered as significant.

### Antibiotic cross resistance

In the final passage in the presence of antibiotic discs, antibiotic cross resistance was determined by testing the MIC of each mutant with all the other antibiotics under study.

### DNA extraction

*B. bacilliformis* were collected by adding sterile PBS into the plates and suspending the colonies with a one-use loop. This bacterial suspension was transferred to a sterile vial and the DNA was extruded by boiling at 100 °C for 10 minutes and stored at −20 °C until use.

### Target mutation detection

The presence of mutations in the *rplV* and *rplD* genes (AZM) as well as in the *23S rRNA* (AZM and CHL), quinolone resistance-determining regions (QRDR) of the *gyrA*, *gyrB*, *parC* and *parE* genes (CIP) and the *rpoB* gene (RIF) were determined by PCR using the primers and conditions listed in Table 6. The PCR products were purified using a commercial kit according to the manufacturer’s instructions (Gel Extraction Kit from Omega Bio-tek, Georgia, USA) and thereafter sequenced (Beckman Coulter, Takeley, United Kingdom). The sequences obtained were compared with those of their respective parental strains and with that of the type strain KC583 (http://www.bacterio.net/bartonella.html). In cases in which the presence of non-silent mutations was observed, the deduced amino acid sequence was compared against *B. bacilliformis* amino acid sequences present in GenBank. Regarding GyrA, hydrophobicity was determined using the method of Kyte and Doolittle as defined in the ProtScale software^60^,^61^, while the polarity pattern was determined using the charge software (http://www.bioinformatics.nl/cgi-bin/emboss/charge).

Throughout the text, both the DNA and amino acid numeration is referred to as that of *B. bacilliformis* KC583. When DNA or amino acid positions of other microorganisms are used for comparison purposes this is explicitly indicated.

### Role of Efflux Pumps

The role of efflux pumps in the development of antibiotic resistance was established by determining the antibiotic susceptibility levels by disc diffusion and E-test in commercial blood agar media supplemented *in house* with PAβN, or ART. In order to determine the EPI concentrations to be used, the effect of different concentrations of these products (from 2.5 mg/L to 20 mg/L) on normal bacterial growth was tested. Additionally, the effect of 100% ethanol (ART solvent) was also independently assessed.

## Additional Information

**How to cite this article**: Gomes, C. *et al*. Development and characterisation of highly antibiotic resistant *Bartonella bacilliformis* mutants. *Sci. Rep.* **6**, 33584; doi: 10.1038/srep33584 (2016).

## Supplementary Material

Supplementary Information

## Figures and Tables

**Table 1:** Number of passages needed to obtain confluent growth.

	Number of passages			
	Strains			
Antibiotic	57.18	57.19	57.20	Mean
Azithromycin	10	12	20	14
Chloramphenicol	>40*	32	40	>37.3
Ciprofloxacin	6	11	15	10.6
Rifampicin	20	4	25	16.3

^*^At the end of 40 passages, in presence of a chloramphenicol disc, a halo of 18 mm was observed.

**Table 2:** MICs and mechanisms of resistance.

Strain					Mechanisms of Resistance									
	MIC^1^ (mg/L)				Fluoroquinolones				Macrolides		Macr + Amph^2^	Rifamycins	EPIs^3^	
	AZM	CHL	CIP	RIF	GyrA	GyrB	ParC	ParE	L4	L22	*23S rRNA*	RpoB	PAβN^4^	ART^5^
57.18	0.19	0.38	0.5	<0.016	wt	wt	Wt	wt	wt	wt	wt	wt	—	—
57.18_Azm-35_	**>256**	**2**	0.75	<0.016	—	—	—	—	Δ_62–65_	wt	wt	—	Y	Y
57.18_Azm-5St_	>256	—	—	—	—	—	—	—	Δ_62–65_	wt	wt	—	—	—
57.18_Chl-40_	<0.016	**4**	0.38	<0.016	—	—	—	—	—	—	wt	—	Y	Y
57.18_Chl-5St_	—	0.125	—	—	—	—	—	—	—	—	wt	—	—	—
57.18_Cip-35_	<0.016	0125	**>32**	<0.016	Val91	wt	Wt	wt	—	—	—	—	N	N
57.18_Cip-5St_	—	—	>32	—	Val91	wt	Wt	wt	—	—	—	—	—	—
57.18_Rif-35_	<0.016	0.75	0.38	**>256**	—				—	—	—	Arg527	N	N
57.18_Rif-5St_	—	—	—	>256	—				—	—	—	Arg527	—	—
57.19	<0.016	0.125	0.38	0.016	wt	wt	Wt	wt	wt	wt	wt	wt	—	—
57.19_Azm-35_	**>256**	**0.5**	0.125	<0.016	—	—	—	—	Arg70	wt	wt	—	N	N
57.19_Azm-5St_	0.5	—	—	—	—	—	—	—	Arg70	wt	wt	—	—	—
57.19_Azm-5St-WH_	32	—	—	—	---	—	—	—	Arg70+Tyr74	wt	wt	—		
57.19_Chl-35_	<0.016	**>256**	0.125	<0.016	—	—	—	—	—	—	A2372	—	N	N
57.19_Chl-5St_	—	0.094	—	—	—	—	—	—	—	—	wt	—	—	—
57.19_Cip-35_	**0.064**	**0.5**	**>32**	<0.016	wt	Lys475	Wt	wt	—	—	—	—	N	N
57.19_Cip-5St_	—	—	1.5	—	wt	Lys475	Wt	wt	—	—	—	—	—	—
57.19_Rif-35_	<0.016	**0.75**	**2**	**>256**	—	—	—	—	—	—	—	Tyr540	N	Y
57.19_Rif-5St_	—	—	—	>256	—	—	—	—	—	—	—	Tyr540	—	—
57.20	0.064	0.25	0.38	<0.016	wt	wt	Wt	wt	wt	wt	wt	wt	—	—
57.20_Azm-35_	**>256**	0.38	**1**	<0.016	—	—	—	—	Lys66	83::VSEAHVGKS	—	—	N	Y
57.20_Azm-5St_	2	—	—	—	—	—	—	—	Lys66*	83::VSEAHVGKS	—	—	—	—
57.20_Chl-40_	<0.016	**>256**	0.25	<0.016	—	—	—	—	—	—	A2372*	—	N	N
57.20_Chl-5St_	—	0.5	—	—	—	—	—	—	—	—	wt	—	—	—
57.20_Cip-35_	0.094	0.25	**>32**	<0.016	Gly95	wt	Wt	wt	—	—	—	—	N	Y
57.20_Cip-5St_	—	—	0.38	—	Gly95*	wt	Wt	wt	—	—	—	—	—	—
57.20_Cip-5St-WH_	—	—	>32	—	Gly95	wt	Wt	wt	—	—	—	—		
57.20_Rif-35_	0.094	**1**	0.047	**>256**	—	—	—	—	—	—	—	Phe545+Tyr588	N	Y
57.20_Rif-5St_	—	—	—	>256	—	—	—	—	—	—	—	Phe545 + Tyr588	—	—

AZM: azithromycin; CHL: Chloramphenicol; CIP: Ciprofloxacin; RIF: Rifampicin.

The asterisks indicate the presence of double peaks, suggesting either the presence of a double bacterial population or the presence of mutations in one of the two copies of the *B. bacilliformis* gene.

Mutants in which cross-resistance to other antibiotics were observed are in underlined font.

The final MIC of the antibiotic used in the mutant selection is shown in bold.

^1^Minimal Inhibitory Concentration.

^2^Macrolides and Amphenicols.

^3^Efflux Pumps Inhibitors.

^4^Phe-Arg-β-Naphtylamide.

^5^Artesunate.

**Table 3:** Cross resistance levels for the mutants obtained.

	MIC 1/ (fold)^2^											
	Azithromycin (AZM)^3^			Chloramphenicol (CHL)^3^			Ciprofloxacin (CIP)^3^			Rifampicin (RIF)^3^		
Ab^4^	57.18_Azm-35_	57.19 _Azm-35_	57.20 _Azm-35_	57.18_Chl-40_	57.19_Chl-35_	57.20_Chl-40_	57.18_Cip-35_	57.19_Cip-35_	57.20_Cip-35_	57.18_Rif-35_	57.19_Rif-35_	57.20_Rif-35_
AZM	—	—	—	**<0.016(<0.08)**	<0.016 (ND)	**<0.016(<0.25)**	**<0.016(<0.08)**	**0.064(>4.00)**	0.094 (1.47)	**<0.016(<0.08)**	<0.016(ND)	0.094 (1.47)
CHL	**2** **(5.26)**	**0.5** **(4.00)**	0.38 ((1.52)	—	—	—	0.125 (0.33)	**0.5** **(4.00)**	0.25 (1.00)	0.75 ((1.97)	**0.75** **(6.00)**	**1** **(4.00)**
CIP	0.75 (1.50)	**0.125** **(0.33)**	**1** **(2.63)**	0.38 ((0.76)	**0.125** **(0.33)**	0.25 ((0.66)	—	—	—	0.38 ((0.76)	**2** **(5.26)**	**0.047** **(0.12)**
RIF	<0.016(ND)	<0.016(<1.00)	<0.016(ND)	<0.016(ND)	<0.016(<1.00)	<0.016(ND)	<0.016(ND)	<0.016(<1.00)	<0.016(ND)	—	—	—

The cases in which the MIC of the mutant decreased with respect to the parental isolate are highlighted in underlined font while those in which the MIC increased, (expressed in mg/L) are shown in bold.

ND: not-determined.

^1^Minimal Inhibitory Concentration in mg/L.

^2^Fold: MIC fold increase/decrease compared to parental isolate. When the quotient ranks between 0.5 and 2 no effect was considered. Values ≥ 2 represent a co-selection of resistance, while values < 0.5 indicate that the mutant strain increased its susceptibility levels to the antimicrobial agent analysed.

^3^Antibiotic used in the selection of resistant mutants.

^4^Antibiotic tested.

**Table 4:** Effect of efflux pump inhibitors (EPIs) on the antibiotic susceptibility levels.

Mutant	Antibiotic			Susceptibility level in presence of EPIs			
		Mutant^1^		PAβN		ART	
		disc^2^	MIC^3^	disc	MIC	disc	MIC
57.18_Azm-35_	AZM	0	>256	**24**	>256	**20**	**48**
57.18_Chl-40_	CHL	18	4	**40**	**1**	**48**	**1.5**
57.18_Cip-35_	CIP	0	>32	0	>32	0	>32
57.18_Rif-35_	RIF	0	>256	0	>256	0	>256
57.19_Azm-35_	AZM	0	>256	0	>256	0	>256
57.19_Chl-35_	CHL	0	>256	0	>256	0	>256
57.19_Cip-35_	CIP	0	>32	0	>32	0	>32
57.19_Rif-35_	RIF	0	>256	0	>256	0	**96**
57.20_Azm-35_	AZM	0	>256	0	>256	**20**	>256
57.20_Chl-40_	CHL	0	>256	0	>256	0	>256
57.20_Cip-35_	CIP	0	>32	0	>32	**20**	>32
57.20_Rif-35_	RIF	0	>256	0	>256	**26**	>256

ART: Artesunate; AZM: azithromycin; CHL: Chloramphenicol; CIP: Ciprofloxacin; RIF: Rifampicin. The samples in which the effect of EPIs was visible highlighted in bold.

^1^Antibiotic susceptibility of mutant isolates at last selection passage.

^2^Disc halo diameter measured in mm.

^3^Minimal inhibitory concentration by E.-test in mg/L.

**Table 5:** Parental isolates characteristics.

Strain			E.D.			Clonal		
CIP	NCTC^1^	Original^1^	Year^1^	Place	Source^1^	AFLP^2^	ISR^2^	Ref.
57.18	12134	267	1949	Lima	Blood	B	2	57
57.19	12135	529	1941	NK	NK	B	2	57
57.20	12136	CPX	1957	NK	Blood	B	2	58

E.D.: Epidemiological data; Original: Name of the original isolate. AFLP: Amplified Fragment Length Polymorphism; ISR: Intergenic Spacer Regions; Ref: Older reference including these strains that we found. NK: Not known.

^1^Information present in http://www.phe-culturecollections.org.uk/.

^2^In Birtles *et al*.^58^.

**Table 6:** Primers used in the study.

Primer	Target	Sequence (5′-3′)	Ann (°C)	Size (bp)	Ref.
Resistance to quinolones					
gyrA-F	*gyrA*	CAT GCG ATG AAT GAA ATG GGA CTT TTG	55	233	19
gyrA-R		AAA CGA CAT TCC GTG TAA CGC ATC GC			
gyrB-F	*gyrB*	CTG AAG TCC GTC CAA TTG TT*	48	634	TS
gyrB-R		TCT TCA AAT GCT GCT TCA TT*			
parE-F	*parE*	CAA TAC GTG ATC CTT TCG AT*	47	564	TS
parE-R		TTC CTC CTT GTG ATA TTC TG*			
parC-F	*parC*	TCT TAT GCT AAG TGT GCA CGG A	55	349	14
parC-R		TAC CAA CAG CAA TCC CTG AAG AA			
Resistance to macrolides					
rplD_F	*rplD*	AGA AGT CTC TGT AGC TGA GGG	49	688	TS
rplD_R		ACT GGA CTG ACA ATT ACA TCA			
rplV_F	*rplV*	CTG GAC TGA CAA TTA CAT CAT	49	702	TS
rplV_R		GGC GAC TCC AAT AGC AGA AG			
Resistance to macrolides and amphenicols					
23S_rRNA_F	*23S rRNA*	AGT GAA ATT GAA TTC CCC	46	780	TS
23S_rRNA_R		GGA ATA CTC GTT TTC AGG T			
Resistance to rifamycins					
rpoB-F	*rpoB*	GAT GAT ATC GAC AAT CTT GGT A**	49	818	TS
rpoB-R		GCA GCA CCT GAA TCA CGA GCC			
Bacterial identification					
16SBartonella-F	*16S rRNA*	CCT TCA GTT MGG CTG GAT C	55	438	59
16SBartonella-R		GCC YCC TTG CGG TTA GCA CA			

TS. This Study.

^*^These primers are modifications of those described by Angelakis *et al*.^43^.

^**^This primer is a modification of the BarpoBF primer designed by Biswas *et al*.^18^.
