# Supplementary material for: Development and characterisation of highly antibiotic resistant Bartonella bacilliformis mutants
Source: Sci Rep. 2016 Sep 26;6:33584. doi: 10.1038/srep33584 (PMC5035977; doi:10.1038/srep33584)

Development and characterisation of highly antibiotic resistant *Bartonella bacilliformis* mutants

Cláudia Gomes[1], Sandra Martínez-Puchol[1†], Lidia Ruiz-Roldán[1‡], Maria J Pons[2], Juana del Valle Mendoza[2,3], Joaquim Ruiz[1*]

[1] ISGlobal, Barcelona Ctr. Int. Health Res. (CRESIB), Hospital Clínic - Universitat de Barcelona, Barcelona, Spain;

[2]School of Medicine, Research Center and Innovation of the Health Sciences Faculty, Universidad Peruana de Ciencias Aplicadas (UPC), Lima, Peru;

[3] Instituto de Investigación Nutricional, Lima, Peru.

Supplementary Figure:

A) Example of double peak showing the presence of a WT and mutated *23S rRNA* sequences (strain 57.20$_{Chl\text{-}40}$)

A2372 (mutated sequence)

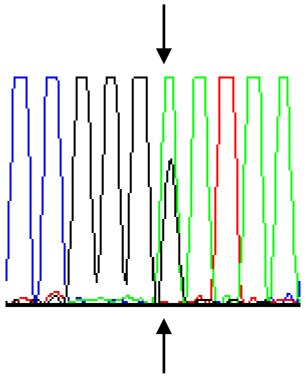

G2372 (wild type sequence)

B) Deletion of 4 amino acids between position 62-65 of L4 (strain 57.18$_{Azm-35}$)

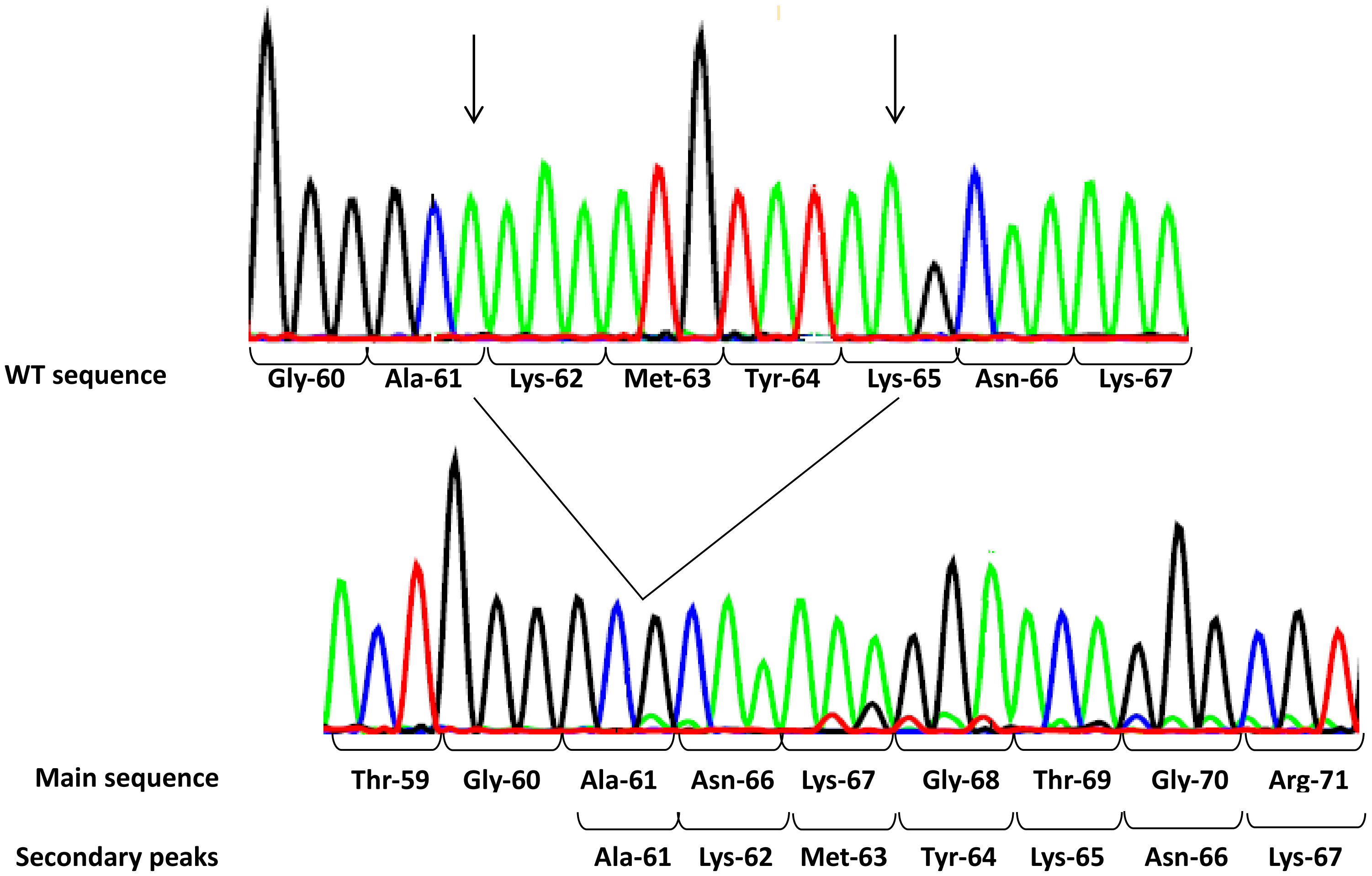

Deletion of 4 amino acids between position 62-65 of L4 (strain 57.18$_{Azm-35}$). In the upper side the DNA sequences belonging to the 57.18 parental isolate, with arrows marking first and the last deleted base in the mutant. In the lower side the sequence of the 57.18$_{Azm-35}$.

In both cases the derived protein sequence is annotated. In addition, the secondary sequence observed under the main sequence is also marked.

C) Duplication of 9 amino acid at position 83 of L22 (strain 57.20$_{Azm}$).

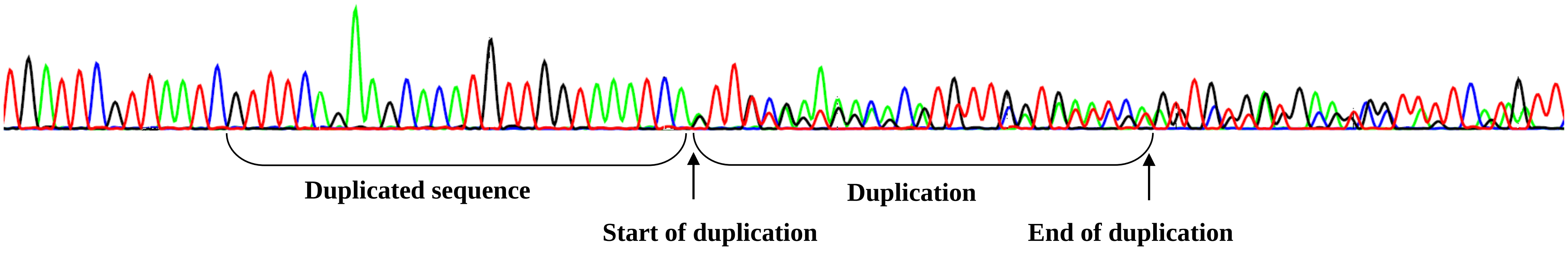

Supplement: Supplementary Information [file srep33584-s1.pdf]
